# First Reported Case of Isolated Persistent Genital Arousal Disorder in a Male

**DOI:** 10.1155/2015/465748

**Published:** 2015-02-12

**Authors:** Bradford J. Stevenson, Tobias S. Köhler

**Affiliations:** Southern Illinois University School of Medicine, 301 N 8th Street-4B, P.O. Box 19665, Springfield, IL 62794, USA

## Abstract

*Introduction*. Persistent genital arousal disorder (PGAD) is a newly recognized disorder in women. It is described as unwanted, persistent feelings of genital arousal unrelated to sexual desire and not relieved by orgasm. Its prevalence is estimated to approach 1% of young women. Until now, this has not been described in men. *Aim*. Here we present a case of a 27-year-old male with symptoms consistent with PGAD and describe successful treatment. *Methods*. A 27-year-old male presented to urology clinic with the chief complain of persistent feelings of impending orgasm. He reported a sensation similar, but not identical, to sexual arousal that did not occur in the setting of sexual thoughts or desire. Orgasm alleviated the arousal for only a short time after which the symptoms would return. This had become quite bothersome to him. *Results*. After assessing for a neurological cause and finding none, the patient was started on paroxetine daily with complete resolution of symptoms. *Conclusions*. PGAD is a disorder previously described only in females. Although symptoms of PGAD have been described in a male as part of another disorder complex, this report describes the first reported isolated case in a male and the successful treatment.

## 1. Introduction

Persistent genital arousal disorder is a newly recognized entity in women. It is described as unwanted, persistent feelings of genital arousal unrelated to sexual desire and not relieved by orgasm. Its prevalence is estimated to approach 1% of young women. Only two cases have been reported in males associated with restless leg syndrome.

## 2. Case Presentation

A 27-year-old male presented to urology clinic with the chief complaint of persistent feelings of impending orgasm. He reported a sensation similar, but not identical, to sexual arousal that did not occur in the setting of sexual thoughts or desire. During these episodes his penis would be extremely sensitive. With manual stimulation he would achieve orgasm, which alleviated the arousal for only a short time after which the symptoms would return. This had become quite bothersome to him.

The onset of symptoms was sudden. This was associated with a change of bowel habits with a sensation of fecal urgency and inability to have a bowel movement without straining. He also complained of an ache in the area of his coccyx. The need to strain resolved with fiber supplementation and did not return, but the fecal urgency persisted. His arousal symptoms persisted as well. He denied any urinary frequency, hesitancy, or urgency. The patient reported normal erections and ejaculation.

His past medical history was significant intermittent neck pain and chronic back pain from a herniated disk at L5-S1, for male pattern baldness, for which he was taking Propecia, and Peyronie's Disease for which he had seen an urologist in the past, but had declined treatment because he was not sexually active. Past surgical history was significant for appendectomy.

Genitourinary exam revealed a circumcised phallus with a 1.5 cm plaque on the dorsum of the penis at the base, otherwise the penile and perineal exam were normal. There was a grade 3 varicocele on the left. No inguinal hernias were present. Digital rectal exam revealed good sphincter tone and a small, smooth, nontender prostate.

The patient was referred to an orthopedic spine surgeon and MRI of the brain and cervical, thoracic, and lumbosacral spinal cord was obtained. The MRI showed the previously diagnosed L5-S1 herniation which was stable and a synovial cyst at L4-5, but no other cysts or abnormalities that would explain the symptoms. He began physical therapy with resolution of his back pain. His arousal, however, persisted.

Having been unsuccessful in finding any obvious neurologic abnormality, the patient was started on paroxetine 20 mg p.o. daily. On follow-up, the patient reported complete resolution of his symptoms. He has since continued on paroxetine without return of symptoms for the past eight years. He has also seen significant improvement in his anxiety level as a result of the medication.

## 3. Discussion

In 2001 Leiblum and Nathan described what they termed persistent sexual arousal syndrome in five women [1]. Since that time, the syndrome has been renamed persistent genital arousal disorder [2] to emphasize that the prominent feature of this disorder is physiologic arousal that is unrelated to and different from normal sexual desire and subjective arousal. This is distinctly different from disorders of hypersexuality which present as excessive sexual desire and compulsive sexual behavior, sometimes referred to as Don Juanism or nymphomania [3]. For a diagnosis of PGAD to be made Goldmeier et al. suggested that the following will be present [4]:

1. symptoms characteristics of sexual arousal (genital fullness/swelling and sensitivity with or without nipple fullness/swelling) that persist for an extended period of time (hours or days) and do not subside completely on their own;
2. symptoms of physiological arousal that do not resolve with ordinary orgasmic experience and may require multiple orgasms over hours or days to remit;
3. symptoms of arousal that are usually experienced as unrelated to any subjective sense of sexual excitement or desire;
4. the persistent genital arousal that may be triggered not only by a sexual activity, but seemingly also by nonsexual stimuli or by no apparent stimulus at all;
5. symptoms that are experienced as unbidden, intrusive, and unwanted;
6. the symptoms that cause the woman at least a moderate degree of distress.

No clear consensus has been reached as to the cause of the disorder. A number of studies have shown that women with PGAD have a higher incidence of psychological disorders such as depression, anxiety, panic attacks, and obsessive compulsive symptoms when compared to non-PGAD women suggesting that there is some psychological basis to the disease. Several studies and case reports have also linked PGAD to antidepressant usage, some having onset of symptoms at the initiation of treatment and some after stopping antidepressants [2, 5].  Other observed associations and theories on the cause of the disorder include dietary, vascular, peripheral, and central neurologic causes and association with restless leg syndrome, although most cases are considered to be idiopathic. A recent review on the subject by Facelle et al. covers the potential etiology in great depth [6]. Since etiology is variable, treatment targeted at the specific cause varies on a case-by-case basis.

Restless genital syndrome (ReGS) is characterized by unpleasant, unwanted genital sensations. It shares several symptoms with PGAD, but the diagnosis requires concomitant restless leg syndrome or overactive bladder symptoms as well as hyperesthesia of the genital region and triggering of the symptoms with manual examination of the ramus inferior to the pubic bone. It is thought to be caused by small fiber sensory neuropathy in genital end branches of the pudendal nerve [7]. This is also a predominantly female syndrome; however, two cases have been described in males [7]. This is the first case of isolated PGAD reported in a male.

## 4. Conclusion

PGAD is a disorder described predominantly in females. Here we present a male patient with symptoms that fulfill the full criteria for the disorder. Although it is uncommon in a male, this report may be an indication that there may be male patients suffering from these symptoms that are being overlooked by providers or who are not seeking help. Our aim in presenting this case is to alert providers and patients to the potential diagnosis of PGAD in a male in the hope that successful treatment may be implemented.

## References

[1] Leiblum S. R., Nathan S. G. (2001). Persistent sexual arousal syndrome: a newly discovered pattern of female sexuality. *Journal of Sex & Marital Therapy*.

[2] Leiblum S. R., Goldmeier D. (2008). Persistent genital arousal disorder in women: case reports of association with anti-depressant usage and withdrawal. *Journal of Sex & Marital Therapy*.

[3] Kafka M. P. (2010). Hypersexual disorder: a proposed diagnosis for DSM-V. *Archives of Sexual Behavior*.

[4] Goldmeier D., Mears A., Hiller J., Crowley T. (2009). Persistent genital arousal disorder: a review of the literature and recommendations for management. *International Journal of STD and AIDS*.

[5] Leiblum S., Seehuus M., Goldmeier D., Brown C. (2007). Psychological, medical, and pharmacological correlates of persistent genital arousal disorder. *Journal of Sexual Medicine*.

[6] Facelle T. M., Sadeghi-Nejad H., Goldmeier D. (2013). Persistent genital arousal disorder: characterization, etiology, and management. *Journal of Sexual Medicine*.

[7] Waldinger M. D., Venema P. L., van Gils A. P. G., de Lint G. J., Schweitzer D. H. (2011). Stronger evidence for small fiber sensory neuropathy in restless genital syndrome: two case reports in males. *Journal of Sexual Medicine*.

